# Supplementary material for: Centriolar satellites are acentriolar assemblies of centrosomal proteins
Source: EMBO J. 2019 Jun 3;38(14):e101082. doi: 10.15252/embj.2018101082 (PMC6627235; doi:10.15252/embj.2018101082)
Supplement: Supplementary file 1 — Appendix [file EMBJ-38-e101082-s001.pdf]

Appendix

Centriolar satellites are acentriolar assemblies of centrosomal proteins

Valentina Quarantotti[1], Jia-Xuan Chen[1#], Julia Tischer[1#], Carmen Gonzalez Tejedo[1], Evaggelia K. Papachristou[1], Clive S. D'Santos[1], John V. Kilmartin[2], Martin L. Miller[1] and Fanni Gergely[1]*

Content

Page 2 Appendix Figures 1-3 and Figure Legends

Page 8 Appendix Supplementary Methods

Page 8 Reagents and Tools Table

Page 11 Methods

Page 18 Appendix Supplementary References

Appendix Figures 1-3 and Figure Legends

Appendix Figure S1: Normalisation of mass-spectrometry data for label-free quantitation. A: Plots showing non-specific binders, such as GFP binders in WT cells and bovine contaminants (excluding immunoglobulins, keratins and α_2-macroglobulin), detected in each pull-down from $WT^{PCM1\text{-}GFP}$ (top panel), STIL-$KO^{PCM1\text{-}GFP}$ (middle panel) and CEP152-$KO^{PCM1\text{-}GFP}$ (bottom panel) cells. All the non-specific binders detected in both the GFP and the IgG control pull-downs within the same replicate are linked by a solid or dashed line and were used for normalisation. Only those non-specific binder pairs showing a $\log_{10}$ LFQ intensity value between 5 and 7.5 (delimited by the black dashed lines) were considered. **B**: Box plot showing the distribution of the data after normalisation, represented as $\log_{10}$ LFQ intensity values, in each paired pull-down, for all the replicates from $WT^{PCM1\text{-}GFP}$ (top panel), STIL-$KO^{PCM1\text{-}GFP}$ (middle panel) and CEP152-$KO^{PCM1\text{-}GFP}$ (bottom panel) cells.

Appendix Figure S1

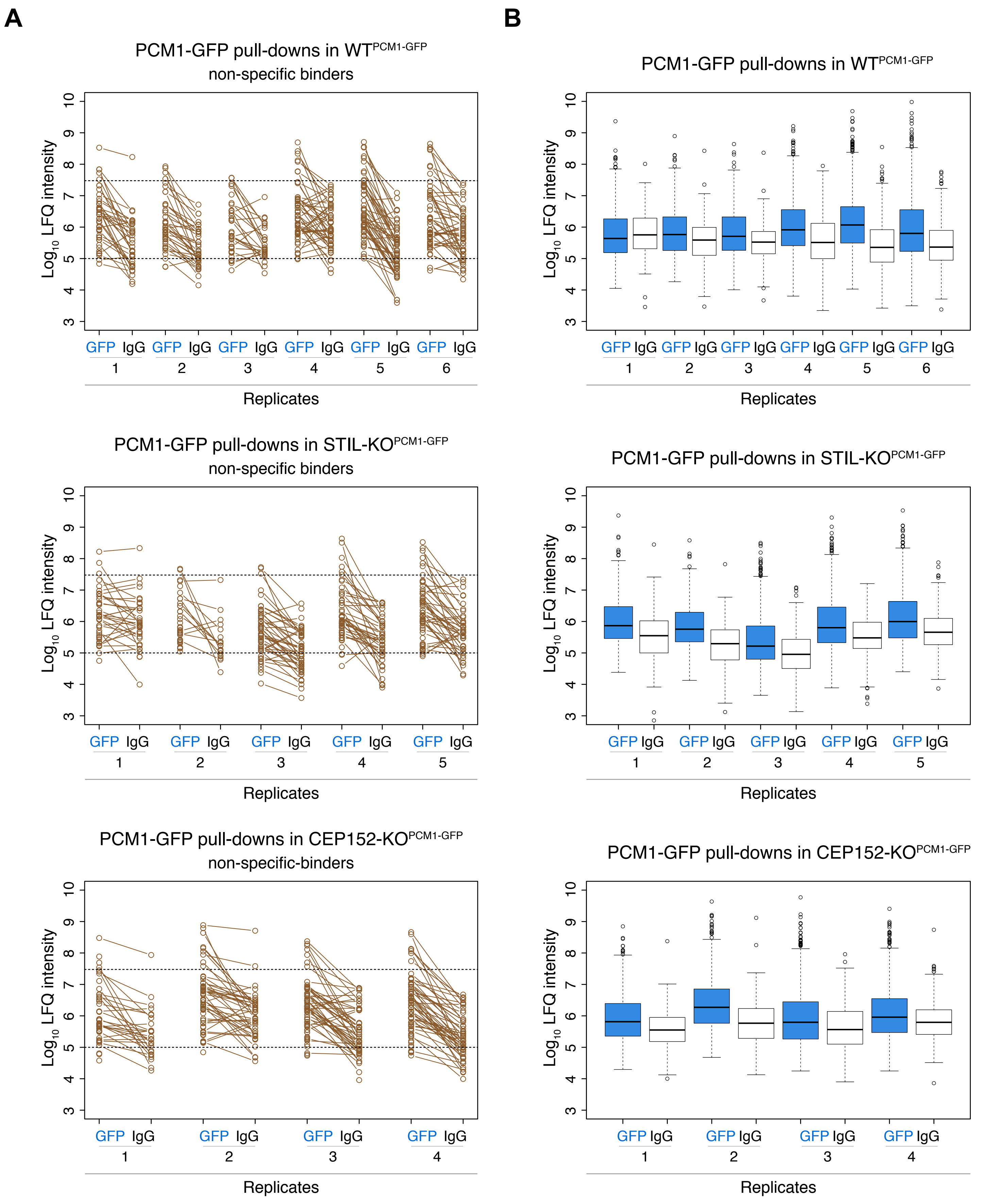

Appendix Figure S2: Characterisation of 293 Flp-In T-REx stable cell lines. Western blots showing the expression of selected centriolar satellite candidates upon induction with increasing concentrations of tetracycline: GFP-CCDC77, GFP-TRIM37 and GFP-CEP170 (**: GFP-tagged protein; *: endogenous protein). In all the western blots, the first lane corresponds to the parental cell line (CON). p150 or α-tubulin were used as loading controls.

Appendix Figure S2

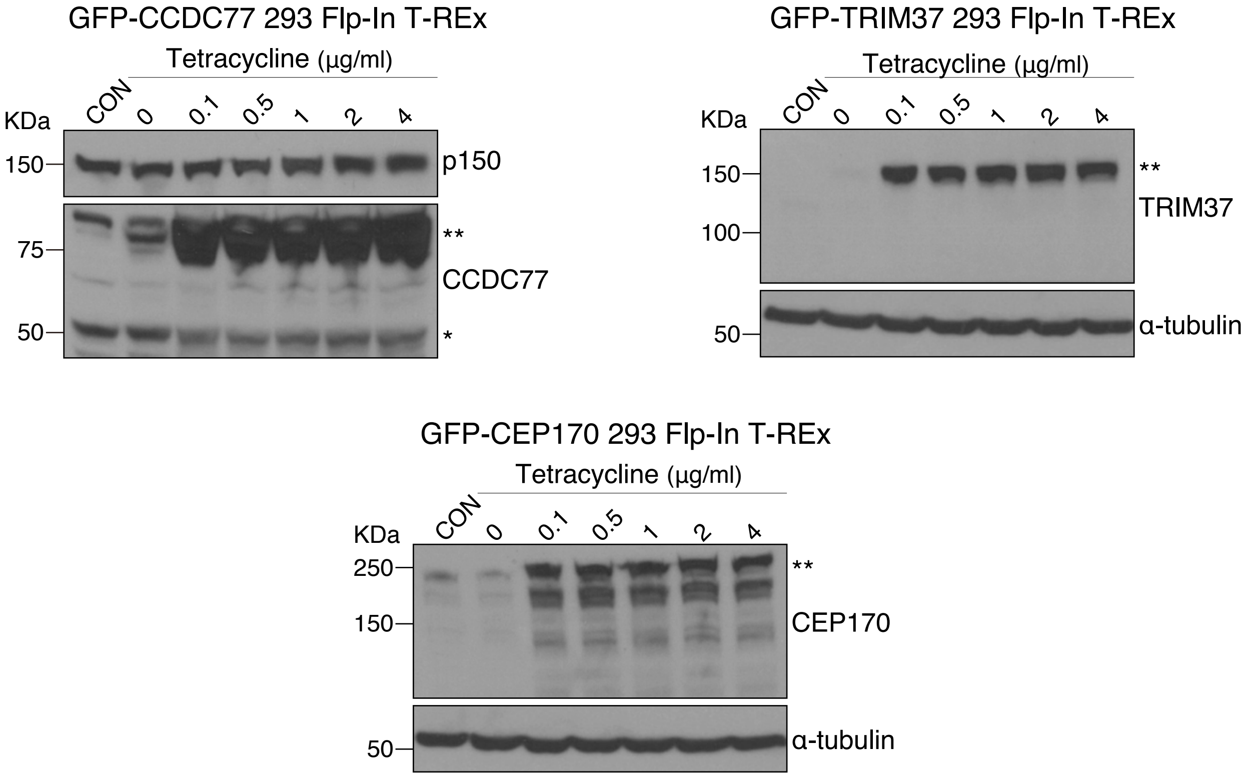

Appendix Figure S3: Differential effects of PCM1 loss on steady-state expression and centrosomal localization of centriolar satellite components. A: Western blots showing the protein levels of selected centrosomal proteins (top panel), microtubule motors and adaptors (middle panel) and kinases (bottom panel) in control (CON) and PCM1-KO clones. The first lane corresponds to the parental cell line (WT). **B**: Box plot showing the signal intensity of select centriolar satellite components at centrosomes of control (CON 1) and PCM1-KO cells (KO 3). The known centriolar satellite components centrin 3 (CETN3), ninein (NIN) and pericentrin (PCNT) were included as positive controls, because their centrosomal accumulation was shown to depend on PCM1 by (Dammermann & Merdes, 2002). Mean signal intensity was determined across centrosomal area defined by γ-tubulin staining. Boxes represent interquartile ranges, whereas whiskers depict 5-95 percentile. Statistical significance was determined using the Mann-Whitney test (**: $p \leq 00.1$; ***: $p \leq 0.001$; ****: $p \leq 0.0001$; n.s.: not significant).

Appendix Figure S3

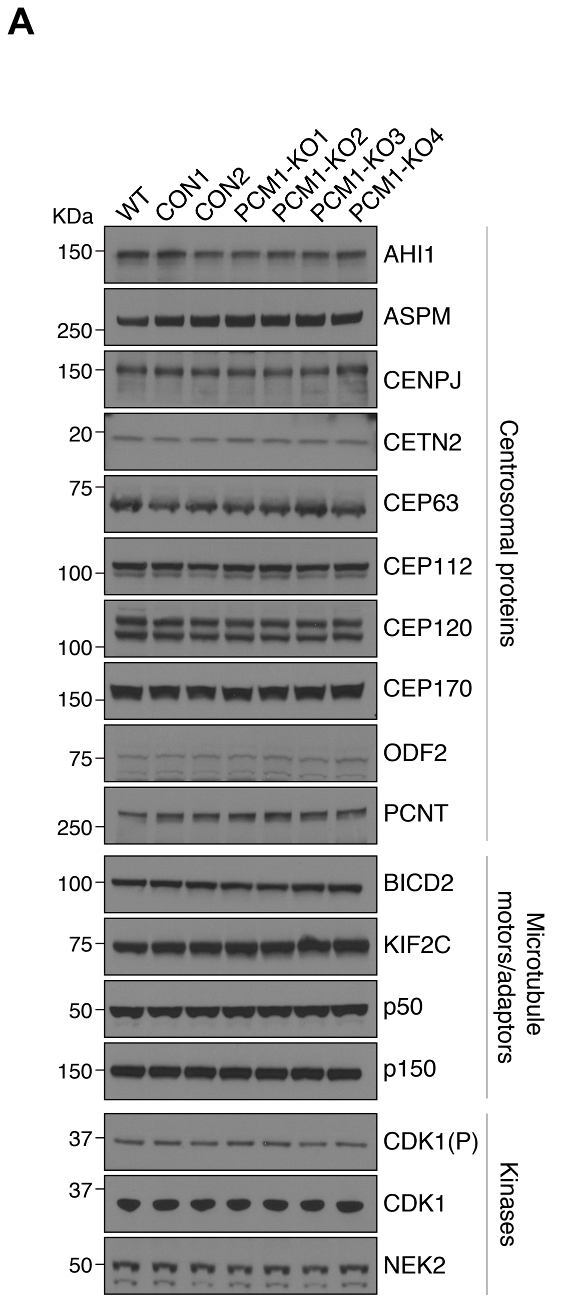

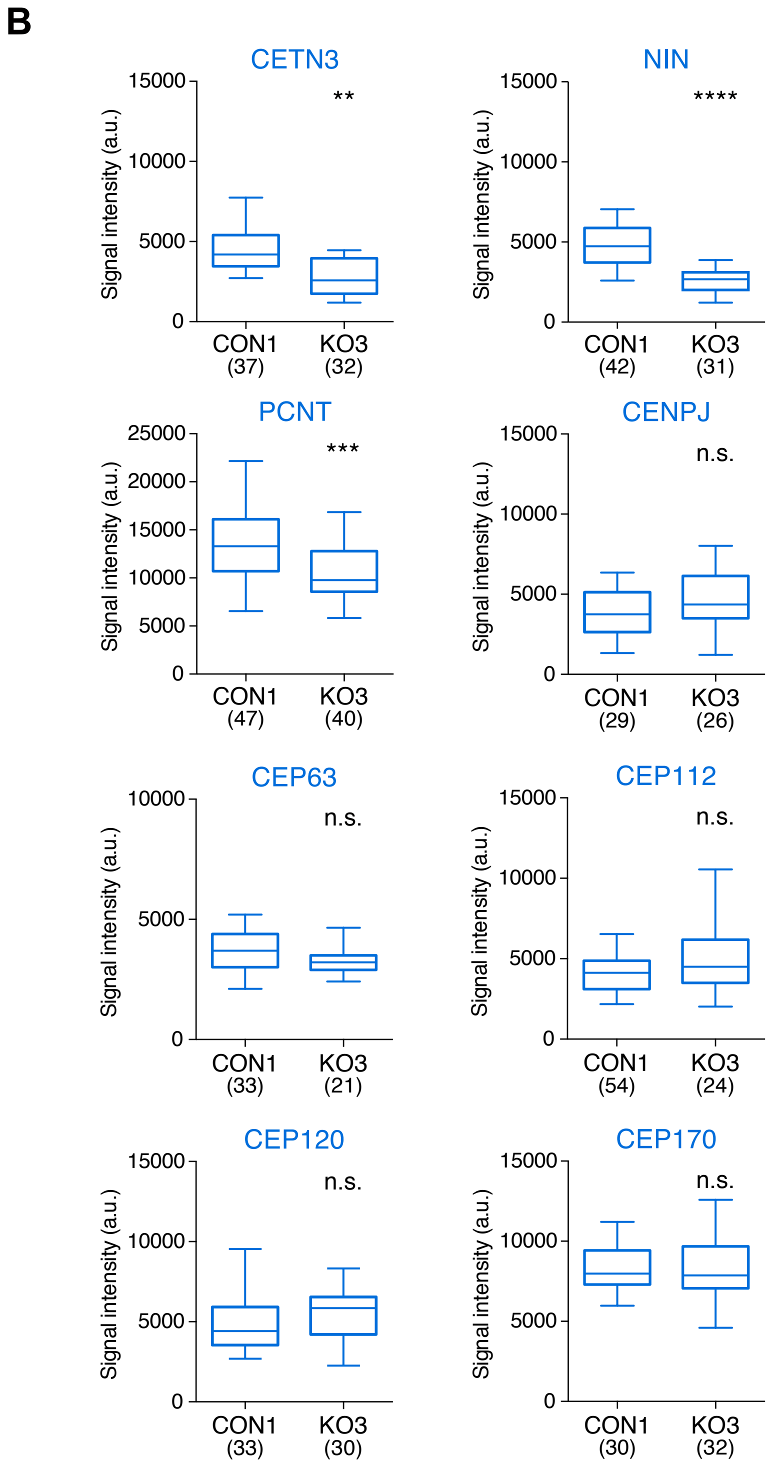

Supplementary Materials and Methods

Reagents and Tools Table

Primary antibodies			
Antibody	Catalogue #	Source	Application
α-tubulin	T9026	Sigma-Aldrich	WB, IF
AHI1	22045-1-AP	Proteintech	WB
ARL13B	17711-1-AP	Proteintech	IF
ASPM	IHC-00058	Bethyl	WB
BBS4	sc-67201	Santa Cruz	IF
BICD2	NBP1-81488	Novus Biologicals	WB, IF
CAPZB	25043-1-AP	Proteintech	WB
CDK1	610037	BD Biosciences	WB
Phospho-CDK1	4539	Cell Signaling	WB
CENPJ	N/A	Gergely lab	WB, IF
CCDC77	HPA038854	Atlas Antibodies	WB
CEP63	N/A	Gergely lab	WB, IF
CEP112	24928-1-AP	Proteintech	WB, IF
CEP120	N/A	Gift by Tsai lab	WB, IF
CEP128	A303-348A	Bethyl	WB, IF
CEP131	ab84864	Abcam	WB, IF
CEP164	22227-1-AP	Proteintech	IF
CEP170	ab72505	Abcam	WB, IF
CEP215	N/A	Gergely lab	WB, IF
CEP290	ab84870	Abcam	WB
CETN2	6288	BioLegend	WB, IF
CETN3	H00001070-M01	Abnova	IF
CP110	12780-1-AP	Proteintech	WB, IF
DCTN1 (p150)	610473	BD Biosciences	WB
DCTN2 (p50)	611002	BD Biosciences	WB
DZIP1	13779-1-AP	Proteintech	WB
DZIP1	AP8926c-EV	Abgent	IF
GFP	11814460001	Roche Diagnostics	WB, IP
GFP	ab6556	Abcam	IF
GFP	ab13970	Abcam	IF
γ-tubulin	T6557	Sigma-Aldrich	WB, IF

HAUS6	HPA020965	Atlas Antibodies	WB
HERC2	612366	BD Biosciences	WB
IgG	I5381	Sigma-Aldrich	IP
IgG	I5006	Sigma-Aldrich	IP
KLHL7	ab90915	Abcam	WB
MCAK	AKIN05	Cytoskeleton	WB
MIB1	EPR2762(2)	GeneTex	WB
MYCBP2	ab86078	Abcam	WB
NEK2	610594	BD Biosciences	WB
NIN	ab4447	Abcam	IF
ODF2	HPA001874	Atlas Antibodies	WB
PCM1	NBP1-87196	Novus Biologicals	WB, IP, IF
PCM1	H00005108-B01P	Abnova	WB, IF
PCNT	ab4448	Abcam	WB, IF
SPICE1	A303-272A	Bethyl	IF
SSX2IP	HPA027306	Atlas Antibodies	WB, IF
TRAF3IP3/T3JAM	sc-398895	Santa Cruz	IF
TRIM37	ab95997	Abcam	WB
WDR37	20916-1-AP	Proteintech	WB
WDR90	HPA049362	Atlas Antibodies	IF
**siRNA sequences**			
siRNA target	siRNA ID	Sequence (5’->3’)	
Control siRNA	4390084	N/A	
BICD2	#1: s23497	AGGUGUGACGAGUACAUUA	
BICD2	#2: s23498	UCUACAACCUGAUCGCUAU	
CCDC77	#1: s38908	GACGUAUCCUGGAAGUAGA	
CCDC77	#2: s38909	GCAUUACCAAAGAGACAUA	
TRIM37	#1: s9081	GGACCGGAGCAGUAUAGAA	
TRIM37	#2: s9082	GACUAGUUAUAUCCAACAA	
TRIM37	#3: s9083	GGUAGUCUAUCACUUCGAA	
TRIM41	#1: s40530	CAAUAGGUGUGAAGAGGUA	
TRIM41	#2: s40532	UGGUCCAGGUGAUUCGGCA	
HERC2	#1: s17062	GCGAAAACAUGGAUGUUCU	
HERC2	#2: s17063	CUAUGGUACGCGAUCGUCA	
WDR37	#1: s225913	GUAGAGAAAUCGACACUCU	

WDR37	#2: s22588	CCUUUAUAUCGAAAACUUA
MYCBP2	#1: s22980	CAUGAUAUGUUUCACCGAA
MYCBP2	#2: s22982	GCCUAAUAAGGUUCGAAAA
CEP170	#1: s19087	GCUGAGAUUUGGAUAUGAU
CEP170	#2: s19088	CCAAAACACCAGAAGGAAA
T3JAM	#1: s37258	GACCAACAAUUAAGAACGA
T3JAM	#2: s37260	GGACCUACAAGAUCAACUA
**Primers used for qPCR reactions**		
Primer ID	Description	Sequence (5'->3')/Catalogue number
GAPDH_qPCR	GAPDH_Fw	CAACAGCCTCAAGATCATCAG
	GAPDH_Rev	ATGGACTGTGGTCATGAGTC
RPS18_qPCR	RPS18_Fw	ATCCCTGAAAAGTTCCAGCA
	RPS18_Rev	CCCTCTTGGTGAGGTCAATG
T3JAM_qPCR	T3JAM_Fw	AGTACTACTGGAGATGGAAGACC
	T3JAM_Rev	TGCTCTGTAGTTGCTGCTCT
BICD2_qPCR	BICD2_Mix	QT00061635 (Qiagen)
CCDC77_qPCR	CCDC77_Mix	QT00091343 (Qiagen)
CEP170_qPCR	CEP170_Mix	QT01675030 (Qiagen)
HERC2_qPCR	HERC2_Mix	QT00068257 (Qiagen)
MYCBP2_qPCR	MYCBP2_Mix	QT00027097 (Qiagen)
TRIM37_qPCR	TRIM37_Mix	QT00052927 (Qiagen)
TRIM41_qPCR	TRIM41_Mix	QT00069132 (Qiagen)
WDR37_qPCR	WDR37_Mix	QT00042952 (Qiagen)

**Primers used to amplify PCM1 cDNA from putative RPE1 PCM1-KO clones**	
Primer ID	Sequence (5'->3')
PCM1_5'UTR_all_isoforms_Fw	AGAGAGTTAATTGTTAAATCCAG
PCM1_5'UTR_all_isoforms_Rev_1	GGTGATCTATTAGATGAGTAAG
PCM1_5'UTR_all_isoforms_Rev_2	CTATTAGATGAGTAAGGCGCTC
PCM1_5'UTR_no_004_Fw	CTCTGCCTTTGACAGGAGAG
PCM1_5'UTR_no_007_Fw	CTGAGCTGCAAAAACTAG
PCM1_Exon_26_all_isoforms_Fw	CAGCCTGGCATCTAAAG
PCM1_Exon_26_all_isoforms_Rev	TAATCCTGCAGGTGCATC

Generation of the 293 Flp-In T-REx cell line expressing GFP fusion products

The 293 Flp-In T-REx cell line (Invitrogen) was a kind gift from Shankar Balasubramanian (Cancer Research UK Cambridge Institute). The pOG44 plasmid and the pCDNA5-FRT-TO vector were gifted by Thomas Mayer (University of Konstanz). The cDNAs of CCDC77 (MHS6278-202829319, Dharmacon), TRIM37 (HsCD00411850, Harvard PlasmID Repository) and CEP170 (MHS6278-213245974, Dharmacon) were inserted in the pCDNA5-FRT-TO vector. Cells were transfected using Lipofectamine 2000 (Invitrogen) according to the manufacturer's instructions. Empty pCDNA5-FRT-TO vector was co-transfected with pOG44 as a control. 10 µg/ml hygromycin-B (InvivoGen) was used to select for stably transfected clones. After 10-15 days single colonies were pooled; to induce transgene expression, 0.1 µg/ml tetracycline (Sigma-Aldrich) was added to cells for 20 hours.

Image processing and analysis

Integrated intensities in fixed cells were measured on maximum intensity projections using the ImageJ multi-measure plugin. To analyse staining intensity of selected candidates at the centrosome, a circle of 2.3 µm diameter was drawn around each centrosome (marked by γ-tubulin) and total intensity was measured across this area. For control and T3JAM siRNAs, total signal intensities of T3JAM were determined within a circle of 8 µm diameter selected to encompass PCM1 staining on maximum intensity projections (Volocity 6.3). Signal intensities were background-corrected. To quantify the number of ciliated cells, nuclei and cilia (based on ARL13B signal) were counted on maximum intensity projections using the multi-point selection tool in Fiji. Cilia length was determined in the same images.

Western blotting

Cells were lysed in RIPA buffer (50 mM Tris-HCl pH 8.0, 150 mM NaCl, 1 mM EDTA, 1 % (v/v) NP-40, 0.5 % (w/v) Na-deoxycholate, 0.1 % (w/v) SDS), supplemented with protease inhibitor cocktail tablets (Complete EDTA-free, Roche Diagnostics). Proteins were separated on NuPAGE pre-cast gels (Invitrogen) and transferred onto the nitrocellulose membrane. Membranes were blocked with 5 % (w/v) milk and 0.1 % (v/v) Tween 20 (Promega) in TBS for 1 hour at room temperature. Primary antibodies (listed in the reagents and tools table) were diluted in blocking solution and incubated overnight at 4 °C. Following 3 x 10 minutes washes in TBS-T, membranes were incubated with Horseradish peroxidase (HRP)-conjugated anti-mouse or anti-rabbit secondary antibodies (DAKO), diluted in blocking solution (1:1000), for 1 hour at room temperature. Following 3 x 10 minutes washes in TBS-T, Pierce ECL Western Blotting Substrates (Thermo Scientific) were used according to the manufacturer's instructions. Chemo-luminescent signal was detected using films.

Homologous gene targeting in DT40 cells

The biallelic tagging of PCM1 with the GFP construct through homologous recombination was achieved by sequentially targeting the two PCM1 alleles using PCM1-GFP constructs containing a different drug selection marker. The PCM1-GFP tagging construct was prepared as a SalI-NotI fragment in pBluescript. First, a SalI-BamHI fragment was prepared by overlapping PCR from DT40 genomic DNA using as a forward oligo (5'-CGCGCGGTCGACTAATTCAGATAA TTGTTACAGCCA-3') from in between exons 31 and 32 (Fig EV1A), incorporating eGFP (referred to as GFP) at the C terminus and ending by placement of a BamHI site 648 bp downstream from the stop codon. The BamHI site was for the addition of drug selection cassettes and was placed here because the closely related Meleagris gallopavo (turkey) sequence has an insertion at this position suggesting this position could accommodate the drug cassette insertion. The right homology arm was a 3.3 kb BamHI-NotI fragment from this BamHI site to the reverse oligo (5'CGCGCGGCGGCCGCGTCGACGTACCTTCCTCCT GGAATTTG-3'), 3.3 kb downstream. The SalI-BamHI and BamHI-NotI fragments were ligated into SalI-NotI pBluescript and checked by sequencing, drug cassettes were added as BamHI fragments. For the sequential targeting, puromycin/Puro and histidinol/His were used in combination with blasticidin/Blasti. The final constructs were linearised using the restriction enzyme NotI HF (NEB). For each transfection, the protocol described by (Barr et al., 2009). Briefly, 1.5×10^7 DT40 cells were collected, washed with ice-cold PBS, resuspended and mixed with 60 μg of the linearised construct, in a total volume of 800 μl of PBS. 24 hours after electroporation using the Biorad Genepulser electroporator, antibiotics were added in order to select for resistant clones at the following concentrations: 50 μg/ml blasticidin (Invitrogen), 0.5 μg/ml puromycin (Sigma-Aldrich) and 1 mg/ml histidinol (Sigma-Aldrich). After 7 to 12 days of selection, resistant clones were picked, expanded and screened for targeted integration of the GFP construct by western blotting. The DT40 cell lines used for endogenous tagging of PCM1 with GFP were the wild-type (WT), STIL knock-out (KO) and CEP152 knock-out (KO) cell lines (Sir et al., 2013).

RNA extraction, cDNA and quantitative Real-Time PCR (qPCR)

RNA extraction was carried out using RNeasy Mini Kit (Qiagen) and the optional DNase digestion (RNase-Free DNase set, Qiagen) was performed, both according to the manufacturer's instructions. The cDNA was prepared from total RNA using the QuantiTect Reverse Transcription Kit (Qiagen), according to the manufacturer's instructions.

Quantitative Real-Time PCR (qPCR) was performed on a QuantStudio 6 Flex Real-Time PCR System (Applied Biosystems), using the Fast SYBR Green Master Mix (Applied Biosystems) and the following thermocycling parameters: 95 °C for 20 seconds, followed by 40 cycles at 95 °C for 1 second and at 60 °C for 20 seconds. *GAPDH* and *RPS18* were the two reference genes used for normalisation of the expression levels. To measure the expression levels relative to control samples, the $2^{-\Delta\Delta CT}$

method (Livak & Schmittgen, 2001) was applied. The primers used for qPCR are listed in the reagents and tools table.

Generation of RPE-1 PCM1 knock-out cell lines by CRISPR/Cas9

The CRISPR/Cas9 based genome editing technology was used to knock-out PCM1 in RPE-1 cells, following the published protocols (Ran et al., 2013). The gRNAs used to target PCM1 were selected using online tools, such as CRISPR Design (www.crispr.mit.edu), CRISPR Search (www.sanger.ac.uk/htgt/wge/ find_crisprs), DESKGEN Cloud (www.deskgen.com) and ChopChop (www.chopchop.cbu.uib.no) were: 5'-caccGATGATCAGGATTTACCAAAC-3', targeting exon 3, and 5'- caccGATCTCGCAAAGCTTCAAACA-3', targeting exon 26. Note that the extra sequences reported in lower case were added to perform the cloning of the gRNAs in the pX458 vector using the BbsI restriction site; in addition, since the gRNAs did not begin with a guanine, an extra nucleotide (underlined) was added, for U6 transcription (Ran et al., 2013). The sense and the antisense oligonucleotides for each gRNA were phosphorylated using the T4 Polynucleotide Kinase (NEB), according to the manufacturer's instructions, and annealed by incubation in boiling water, which was then left to cool-down at room temperature for ~ 2 hours. Then, they were ligated into the BbsI digested pX458 plasmid (#48138, Addgene) previously digested with the BbsI restriction enzyme (NEB), using the Quick Ligation Kit (NEB), according to the manufacturer's instructions. To verify the correct insertion of the gRNAs into pX458, plasmids were sequenced by Sanger sequencing.

RPE-1 cells were seeded the day before transfection, to achieve ~ 70 % confluency on the day of transfection. Cells were transfected using Viromer RED (Lipocalyx GmbH), according to the manufacturer's instructions. The pX458 vector without gRNA was used as a control. 48 hours after transfection, the GFP-positive cells were single sorted into 96 well plates by FACS, using BD FACSAria IIU (BD Biosciences). Single clones were expanded and screened by western blotting to identify putative PCM1 knock-out clones.

To identify the DNA sites of the CRISPR/Cas9-mediated cleavage and to further characterize the transcript variants, the gRNA-targeted sequences were amplified using cDNA as a template (~ 200 ng) (primers are listed in the reagents and tools table). The blunt-ended PCR products obtained using the using Phusion High-Fidelity DNA polymerase (Thermo Scientific), according to the manufacturer's instructions, were gel extracted and cloned into the pJET1.2/blunt vector, using the CloneJET PCR Cloning Kit (Thermo Fisher Scientific), following the manufacturer's instructions. Gel extraction of PCR products was performed using PureLink Quick Gel Extraction Kit (Invitrogen), according to the manufacturer's instructions. 10 to 20 single bacterial colonies per cDNA were analysed by Sanger sequencing.

Gel staining

Coomassie staining was performed by incubating the gel with the InstantBlue solution (Expedeon) for 4 hours at room temperature. Silver staining was performed according to the method described by Shevchenko et al. (1996). Briefly, the gel was rinsed with Milli-Q water and fixed in the fixative solution (45 % (v/v) methanol and 5 % (v/v) acetic acid in Milli-Q water) overnight at 4 °C, while shaking. Then, the gel was rinsed with Milli-Q water for at least 1 hour and sensitized by incubation for 10 minutes in 0.02 % (w/v) Na-thiosulphate. Following 2 x 1 minute washes in Milli-Q water, the gel was incubated in the silver solution (0.1 % (v/v) silver nitrate (Thermo Fisher Scientific)) for 40 minutes at 4 °C. Afterwards, the gel was rinsed 2 x 1 minute in Milli-Q water and developed with the silver stain developer solution (20 g/l Na_2CO_3-0.4 g/l paraformaldehyde). Pouring off the developing solution and adding 1 % (v/v) acetic acid stopped the reaction. The gel was then imaged using the Image Scanner III (GE Healthcare Life Sciences).

Sample preparation and liquid chromatography tandem mass spectrometry (LC-MS/MS)

For label-free and SILAC CS isolation experiments (Figs 2 and 6), coomassie stained gel lanes were "in-gel" digested: gel bands were excised from each lane using a GridCutter (The Gel Company) and to increase the efficiency of the tryptic digestion, each band was further cut into 4 pieces, which were transferred into the same 1.5 ml tube. The gel pieces were shrunk with 150 µl Acetonitrile (MeCN) (Optima LC/MS Grade, Fisher Chemical) and incubated at room temperature for 15 minutes, while shaking (at 850 rpm). Then, the MeCN solution was removed and the gel pieces were rehydrated with 150 µl 100 mM ammonium bicarbonate (AmBic) (Fisher Chemical), pH 8.5. After incubating at room temperature for 15 minutes, while shaking, the washes with MeCN and AmBic were repeated once. The gel pieces were shrunk again in MeCN and then incubated with 150 µl of reducing solution (10 mM DTT (Fisher Chemical) in 100 mM AmBic, pH 8.5) at 56 °C for 60 minutes, while shaking. Then, the reducing solution was removed and the gel pieces washed once with 150 µl of Iodoacetamide solution (100 mM Iodoacetamide (ACROS Organics) in 100 mM AmBic, pH 8.5). To alkylate the proteins, 150 µl of Iodoacetamide solution were added to the gel pieces and incubated for 45 minutes at room temperature in the dark. Then, the gel pieces were shrunk with MeCN, washed with AmBic and shrunk again as described above, before proceeding with the tryptic digestion. Samples were digested overnight at 37 °C, by incubation with 150 ng of Trypsin (Worthington Biochemical) in 100 mM AmBic. After the overnight proteolysis, the supernatants were transferred to new tubes and 50 µl of 5 % (v/v) Pierce Formic Acid, LC-MS Grade (Thermo Fisher Scientific) in MeCN was added to the gel pieces. The incubation was carried out for 15 minutes at room temperature, while shaking; then the supernatants from the gel pieces were removed and combined with the original digest. The peptide solutions were then dried using the Speedvac Concentrator 5301 (Eppendorf).

Peptides (6.5 µl for each group of 4 "in-gel" digested pooled bands) were first loaded onto a C18 trap column (Acclaim PepMap 100, inner diameter: 100 µm, length: 2 cm, particle size: 5 µm, pore size:

100 Å) and then resolved on a 25 cm C18 analytical column (Acclaim PepMap RSLC, inner diameter: 75 µm, particle size: 2 µm, pore size: 100 Å) through a 55 minutes linear gradient of 4-32% Acetonitrile (MeCN) (Optima LC/MS Grade, Fisher Chemical) plus 0.1 % Formic Acid and 5 % DMSO at a constant flow rate of 300 nl/min. Eluted peptides were transferred directly by electrospray ionization at 2.5 kV into the LTQ Orbitrap Velos mass spectrometer (Thermo Scientific).

Mass spectra were acquired in data-dependent mode using a "top20" method. Each duty cycle consisted of one MS full scan in the Orbitrap mass analyser with real-time internal calibration at lock mass of 445.1200 m/z (resolution: 60,000, target value: 1×10^6, scan range: 400 to 1,600 m/z) and subsequent MS/MS scans of 20 most intense precursor ions fragmented via collision-induced dissociation in the linear trap quadrupole (LTQ; target value: 1×10^4, isolation window: 2.0 m/z, normalised collision energy: 30 %, wideband activation enabled). Precursor ions with unassigned or + 1 charge state were not selected for fragmentation scans. In addition, precursor ions already isolated for fragmentation were dynamically excluded for 30 seconds.

Each SILAC whole cell proteome sample (380 µg) was trypsin-digested and subjected to high-pH reversed-phase fractionation. The separated peptides were concatenated into 20 fractions. Samples were measured by a Q Exactive or Q Exactive HF Orbitrap mass spectrometer; each fraction was measured using a 120-min top10 method consisting of a full scan (resolution: 35,000 or 60,000, target value: 3×10^6, scan range: 400 to 1,600 m/z) and subsequent MS/MS scans of 10 most intense precursor ions fragmented via higher energy collisional dissociation (HCD; target value: 17,500 or 30,000, isolation window: 2.0 m/z, normalised collision energy: 28%).

MaxQuant software data processing and bioinformatic data analysis

Mass spectrometry raw data files were processed using MaxQuant software (version 1.5.3.30 or 1.6.1.0) (Cox & Mann, 2008). MS/MS mass spectra were searched using Andromeda search engine (Cox et al., 2011) against a target-decoy database containing the forward and reverse protein sequences of UniProt *G. gallus* proteome release 2017_03 (29,733 entries), Avian leukosis virus-RSA (3 entries; retrieved on 27 July 2016 from NCBI), GFP and the default list of 245 common contaminants. *In silico* digestion of the proteome database was performed based on trypsin/P specificity (cleave C-terminally to arginine or lysine residues even if followed by proline). A maximum of 2 missed cleavages were allowed. Carbamidomethylation of cysteine was set as fixed modification. Both methionine oxidation and protein N-terminal acetylation were considered as variable modifications. Minimum peptide length was 7 amino acids. At least one unique peptide was required for each protein group. "Second peptides" option was activated. "Match between runs" option was switched on to allow transfer of identifications in a time window of 0.7 minutes between same/adjacent fractions of pull-down samples (but not between replicates of the label-free pull-downs). False discovery rate (FDR) was set to 1 % at both peptide and protein levels. For protein quantification, both unique and razor peptides were included (Nesvizhskii & Aebersold, 2005).

Label-free quantification was performed using the MaxLFQ algorithm (Cox et al., 2014) with the following settings: minimum ratio count of one; fast LFQ deactivated; normalisation skipped. For the SILAC pulldown experiments, heavy labels (Arg-10, Lys-8) were assigned to identify SILAC peptide pairs. A maximum of 3 SILAC-labelled amino acids were allowed for each peptide. For SILAC protein quantification, minimum ratio count was set to one and the “advanced ratio estimation” option was activated. The “re-quantify” function was also switched on.

All data analyses were performed in R statistical environment (version 3.4.0) unless otherwise stated. For the label-free data normalisation, in order to normalise the LFQ intensity values between each pair of anti-GFP and IgG control pull-downs, a list of non-specific binders was first constructed. These non-specific binders consisted of the detected bovine contaminants (excluding keratins, immunoglobulins and α_2-macroglobulin) and the proteins detected in a separate anti-GFP pull-down with untagged DT40 cells. We further limited this list of non-specific binders with an LFQ intensity filter (lower limit: 1×10^5; upper limit: 3×10^7) to prevent over-correction. Under the assumption that the non-specific background proteins should bind equally to the anti-GFP beads as well as the IgG controls, we calculated the mean difference in log-transformed LFQ intensities of these background proteins between the anti-GFP and IgG pull-downs. This mean difference was then used as a global normalisation factor to adjust LFQ intensity values. The normalisation procedure was performed individually for each pair of anti-GFP and IgG control pull-downs.

To detect PCM1 specific interactors, data were first filtered to retain proteins that had been detected in at least 4/6 ($\mathrm{WT}^{\mathrm{PCM1\text{-}GFP}}$), 3/5 ($\mathrm{STIL\text{-}KO}^{\mathrm{PCM1\text{-}GFP}}$) or 3/4 ($\mathrm{CEP152\text{-}KO}^{\mathrm{PCM1\text{-}GFP}}$) of the pull-down replicates in either anti-GFP or IgG control groups. Using the Perseus software (version 1.5.5.0) (Tyanova et al., 2016), we replaced the missing values by randomly picking from a normal distribution (down shift = 1.8; width = 0.3) that simulates low abundant proteins below the detection limit for each pull-down. The enrichment and its significance (paired samples Student’s *t* test) of proteins detected in the GFP versus IgG control pull-downs were then plotted in a volcano plot. Specific interactions were determined using a combined threshold based on the SAM (Significance Analysis of Microarrays) algorithm ($s_0 = 1.5$; $t_0 = 1.4$) (Li, 2012, Tusher et al., 2001). In PRIDE partner repository the MaxQuant output protein groups file is named “proteinGroups.txt”; the normalised unfiltered dataset is “prot_untagged_binder_contaminant_pairwise_mean_corrected orthologues.txt” and the filtered datasets are “WT_output_SAM_orthologues_bioid_centrosome.txt”, “STIL_output_SAM orthologues_centrosome.txt” and “CEP152_output_SAM_orthologues_centrosome.txt”.

Orthologous gene mapping between chicken and human was performed based on orthology information provided by Ensembl (Herrero et al., 2016). Conversion to Entrez gene identifiers was done in Bioconductor (Gentleman et al., 2004). Gene ontology (GO) analysis of chicken data was carried out using the converted human orthologous genes. For GO enrichment analysis, the background gene list was compiled by converting the *G. gallus* proteome to human orthologous genes. Using the GOstats package (Falcon & Gentleman, 2007), a conditional hypergeometric test was performed to

assess the significance of enrichment, followed by false discovery rate control with the Benjamini-Hochberg approach (Benjamini & Hochberg, 1995).

In the whole cell proteome SILAC experiments, persistently "light"-labelled contaminant proteins across label-swap replicates were filtered out. Differentially-regulated proteins in SILAC experiments were determined by Significance A/B (Cox & Mann, 2008), calculated using the Perseus software.

Supplementary References

Barr AR, Zyss D, Gergely F (2009) Knock-in and knock-out: the use of reverse genetics in somatic cells to dissect mitotic pathways. *Methods Mol Biol* 545: 1-19

Benjamini Y, Hochberg Y (1995) Controlling the false discovery rate: a practical and powerful approach to multiple testing. *J R Stat Soc Ser B-Methodol* 57: 289-300

Cox J, Hein MY, Luber CA, Paron I, Nagaraj N, Mann M (2014) Accurate proteome-wide label-free quantification by delayed normalization and maximal peptide ratio extraction, termed MaxLFQ. *Mol Cell Proteomics* 13: 2513-26

Cox J, Mann M (2008) MaxQuant enables high peptide identification rates, individualized p.p.b.-range mass accuracies and proteome-wide protein quantification. *Nat Biotechnol* 26: 1367-72

Cox J, Neuhauser N, Michalski A, Scheltema RA, Olsen JV, Mann M (2011) Andromeda: a peptide search engine integrated into the MaxQuant environment. *J Proteome Res* 10: 1794-805

Falcon S, Gentleman R (2007) Using GOstats to test gene lists for GO term association. *Bioinformatics* 23: 257-8

Gentleman RC, Carey VJ, Bates DM, Bolstad B, Dettling M, Dudoit S, Ellis B, Gautier L, Ge Y, Gentry J, Hornik K, Hothorn T, Huber W, Iacus S, Irizarry R, Leisch F, Li C, Maechler M, Rossini AJ, Sawitzki G et al. (2004) Bioconductor: open software development for computational biology and bioinformatics. *Genome Biol* 5: R80

Herrero J, Muffato M, Beal K, Fitzgerald S, Gordon L, Pignatelli M, Vilella AJ, Searle SM, Amode R, Brent S, Spooner W, Kulesha E, Yates A, Flicek P (2016) Ensembl comparative genomics resources. *Database (Oxford)* 2016

Li W (2012) Volcano plots in analyzing differential expressions with mRNA microarrays. *J Bioinform Comput Biol* 10: 1231003

Livak KJ, Schmittgen TD (2001) Analysis of relative gene expression data using real-time quantitative PCR and the 2(-Delta Delta C(T)) Method. *Methods* 25: 402-8

Nesvizhskii AI, Aebersold R (2005) Interpretation of shotgun proteomic data: the protein inference problem. *Mol Cell Proteomics* 4: 1419-40

Ran FA, Hsu PD, Wright J, Agarwala V, Scott DA, Zhang F (2013) Genome engineering using the CRISPR-Cas9 system. *Nature protocols* 8: 2281-2308

Shevchenko A, Wilm M, Vorm O, Mann M (1996) Mass spectrometric sequencing of proteins silver-stained polyacrylamide gels. *Anal Chem* 68: 850-8

Tusher VG, Tibshirani R, Chu G (2001) Significance analysis of microarrays applied to the ionizing radiation response. *Proc Natl Acad Sci U S A* 98: 5116-21

Tyanova S, Temu T, Sinitcyn P, Carlson A, Hein MY, Geiger T, Mann M, Cox J (2016) The Perseus computational platform for comprehensive analysis of (prote)omics data. *Nat Methods* 13: 731-40
