# Supplementary material for: Centriolar satellites are acentriolar assemblies of centrosomal proteins
Source: EMBO J. 2019 Jun 3;38(14):e101082. doi: 10.15252/embj.2018101082 (PMC6627235; doi:10.15252/embj.2018101082)
Supplement: Supplementary file 2 — Expanded View Figures PDF [file EMBJ-38-e101082-s002.pdf]

Expanded View Figures

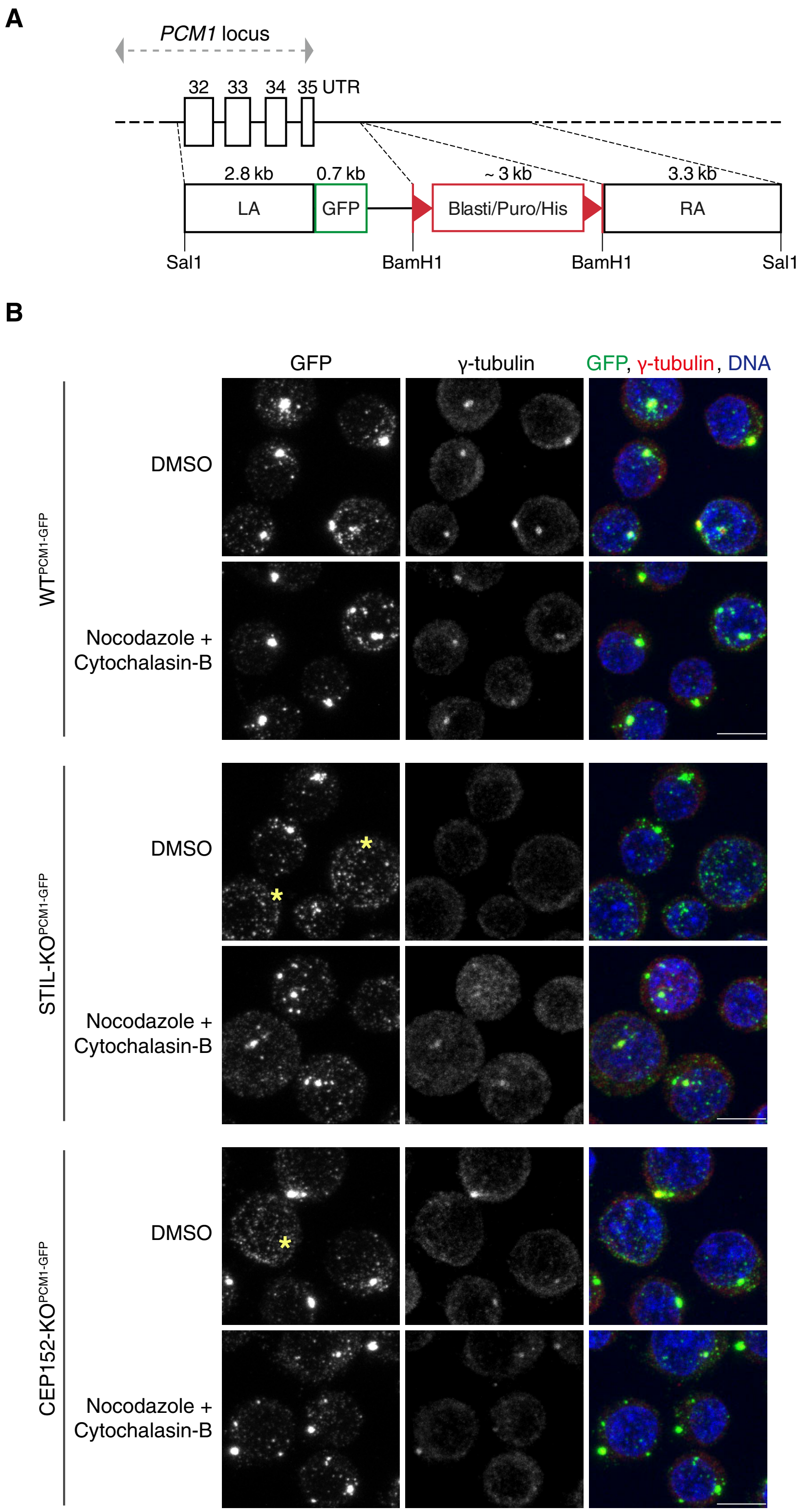

Figure EV1. The effect of combined nocodazole and cytochalasin-B treatment on the distribution of endogenously labelled PCM1-GFP in DT40 cells.

A Diagram showing the GFP construct used to target the chicken *PCM1* locus at the C-terminus on both alleles, by homologous recombination. Highlighted the Sal1 and BamH1 sites used for restriction digestion to clone the LA (Left Arm) and the RA (Right Arm) and to replace the resistance cassette. Clones were screened for antibiotic resistance genes blasticidin (Blasti), puromycin (Puro) or Histidinol (His). LoxP sites flanking the resistance cassette are represented by red triangles. The dashed lines indicate the sites of recombination and integration in the *PCM1* locus. Confirmation of targeting was carried out by Western blotting, as shown in Fig 1B–D.

B Representative immunofluorescence images of cell lines with genotypes as indicated, treated with both nocodazole (2 μg/ml) and cytochalasin-B (1 μg/ml). DMSO-treated cells were used as a control (DMSO, upper panels). Treatments were carried out for 2 h, and cells were co-stained with antibodies against GFP (green) and γ-tubulin (red). DNA is in blue. Images correspond to maximum intensity projections of confocal micrographs. Asterisks mark cells with dispersed satellites. Note that drug treatment leads to an increase in large and a decrease in small satellite granules in all three genotypes, but the effects are more prominent in acentriolar than in WT cells. Scale bars: 5 μm.

© 2019 The Authors

Figure EV2. Comparisons of CS-WT with published datasets.

A Venn diagram showing the number of proteins identified in three datasets: CS-WT, PCM1-BioID (Gupta *et al*, 2015) and PCM1-FLAG IP (Gupta *et al*, 2015). Note that this and all subsequent analyses were performed on human orthologues of the chicken proteins from CS-WT.

B Venn diagrams showing the number of published CS components (illustrated in the table in C) detected in each of the indicated datasets.

C Table depicts previously reported CS components and their detection in the indicated datasets.

D Venn diagram showing the number of centrosomal proteins based on the human centrosome proteome dataset (Jakobsen *et al*, 2011) detected in each of the indicated datasets. Note that the majority of the proteins detected in both CS-WT and PCM1-BioID are centrosomal proteins (33/43).

E Venn diagram showing the overlap between proteins identified in CS-WT and in a functional screen for positive regulators of Hedgehog signalling (Breslow *et al*, 2018).

© 2019 The Authors

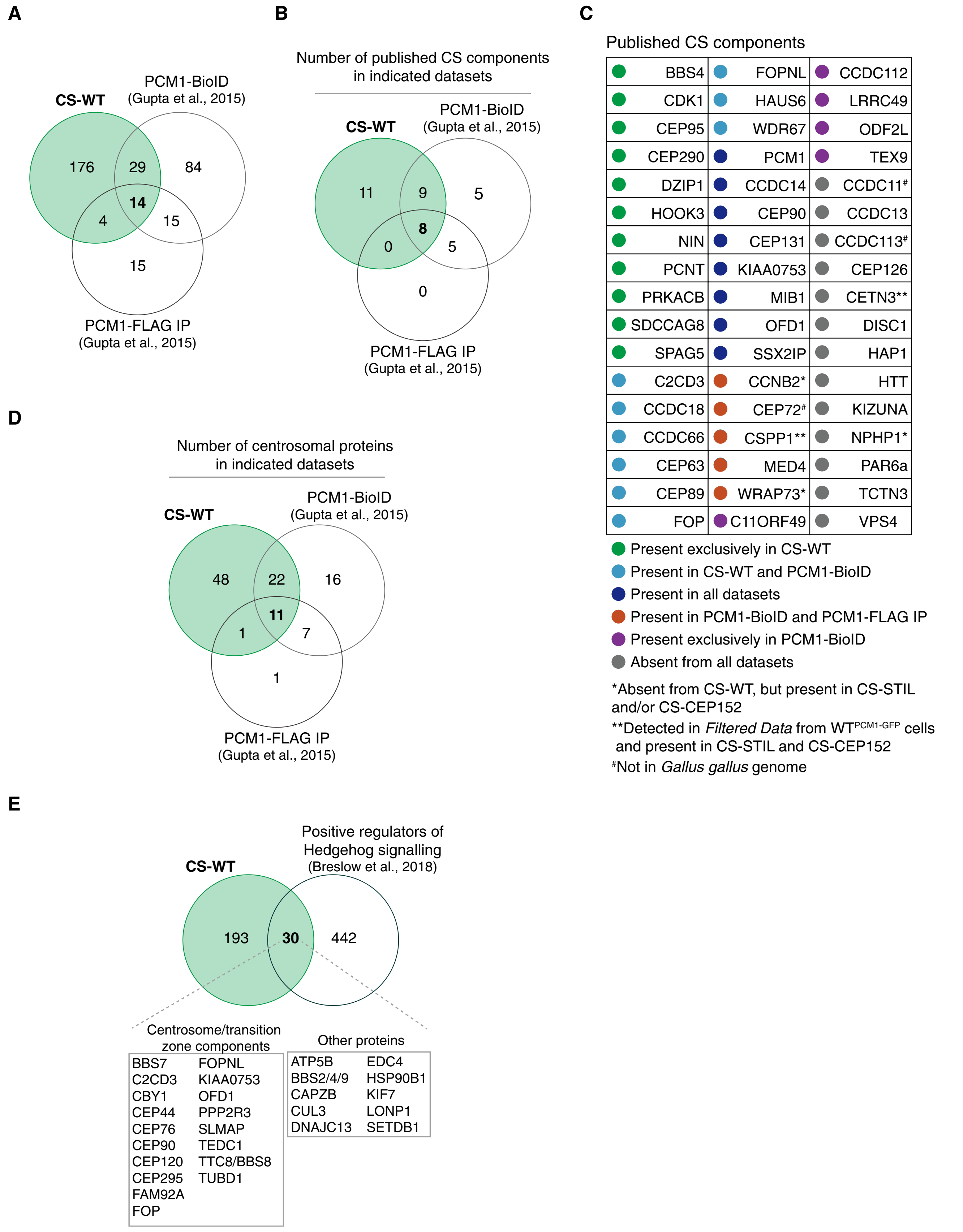
A
CS-WT
PCM1-BioID
(Gupta et al., 2015)
176
29
84
14
4
15
15
PCM1-FLAG IP
(Gupta et al., 2015)
B
Number of published CS components
in indicated datasets
CS-WT
PCM1-BioID
(Gupta et al., 2015)
11
9
5
8
0
5
0
PCM1-FLAG IP
(Gupta et al., 2015)
C
Published CS components
BBS4
CDK1
CEP95
CEP290
DZIP1
HOOK3
NIN
PCNT
PRKACB
SDCCAG8
SPAG5
C2CD3
CCDC18
CCDC66
CEP63
CEP89
FOP
FOPNL
HAUS6
WDR67
PCM1
CCDC14
CEP90
CEP131
KIAA0753
MIB1
OFD1
SSX2IP
CCNB2*
CEP72#
CSPP1**
MED4
WRAP73*
C11ORF49
CCDC112
LRRC49
ODF2L
TEX9
CCDC11#
CCDC13
CCDC113#
CEP126
CETN3**
DISC1
HAP1
HTT
KIZUNA
NPHP1*
PAR6a
TCTN3
VPS4
Present exclusively in CS-WT
Present in CS-WT and PCM1-BioID
Present in all datasets
Present in PCM1-BioID and PCM1-FLAG IP
Present exclusively in PCM1-BioID
Absent from all datasets
*Absent from CS-WT, but present in CS-STIL and/or CS-CEP152
**Detected in Filtered Data from WT PCM1-GFP cells and present in CS-STIL and CS-CEP152
#Not in Gallus gallus genome
D
Number of centrosomal proteins
in indicated datasets
CS-WT
PCM1-BioID
(Gupta et al., 2015)
48
22
16
11
1
7
1
PCM1-FLAG IP
(Gupta et al., 2015)
E
CS-WT
Positive regulators of
Hedgehog signalling
(Breslow et al., 2018)
193
30
442
Centrosome/transition
zone components
BBS7
C2CD3
CBY1
CEP44
CEP76
CEP90
CEP120
CEP295
FAM92A
FOP
FOPNL
KIAA0753
OFD1
PPP2R3
SLMAP
TEDC1
TTC8/BBS8
TUBD1
Other proteins
ATP5B
BBS2/4/9
CAPZB
CUL3
DNAJC13
EDC4
HSP90B1
KIF7
LONP1
SETDB1

Figure EV2.

Figure EV3. Localisation of centriolar satellite candidates in Jurkat cells.

A Representative immunofluorescence images of Jurkat cells co-stained with antibodies against selected new CS candidates (green) and PCM1 (red). SSX2IP, a known CS component, is shown as positive control. The framed panel at the bottom illustrates the relative distributions of the centrosomal marker γ-tubulin (red) and the CS protein PCM1 (green) in Jurkat cells. DNA is in blue. Images correspond to maximum intensity projections of confocal micrographs. High magnification images are included to aid visualisation of framed areas. Scale bars: 5 μm.

B Representative immunofluorescence images of Jurkat cells mock-treated with DMSO or incubated with nocodazole (2 μg/ml) to depolymerise microtubules. Cells were co-stained with antibodies against PCM1 (green), γ-tubulin (red) and α-tubulin (blue). Nocodazole reduces PCM1 signal in the pericentrosomal region. High magnification images are included to aid visualisation of framed areas and correspond to framed areas. Scale bar: 5 μm.

C Knock-down efficiency of siRNAs assessed by qPCR. The relative expression of each candidate upon siRNA treatment was assessed by qPCR and shown relative to cells treated with a control siRNA. Each datapoint represents a biological replicate. Note that T3JAMsi1 enhances rather than reduces mRNA expression. Bar graphs show mean ± SE.

 © 2019 The Authors

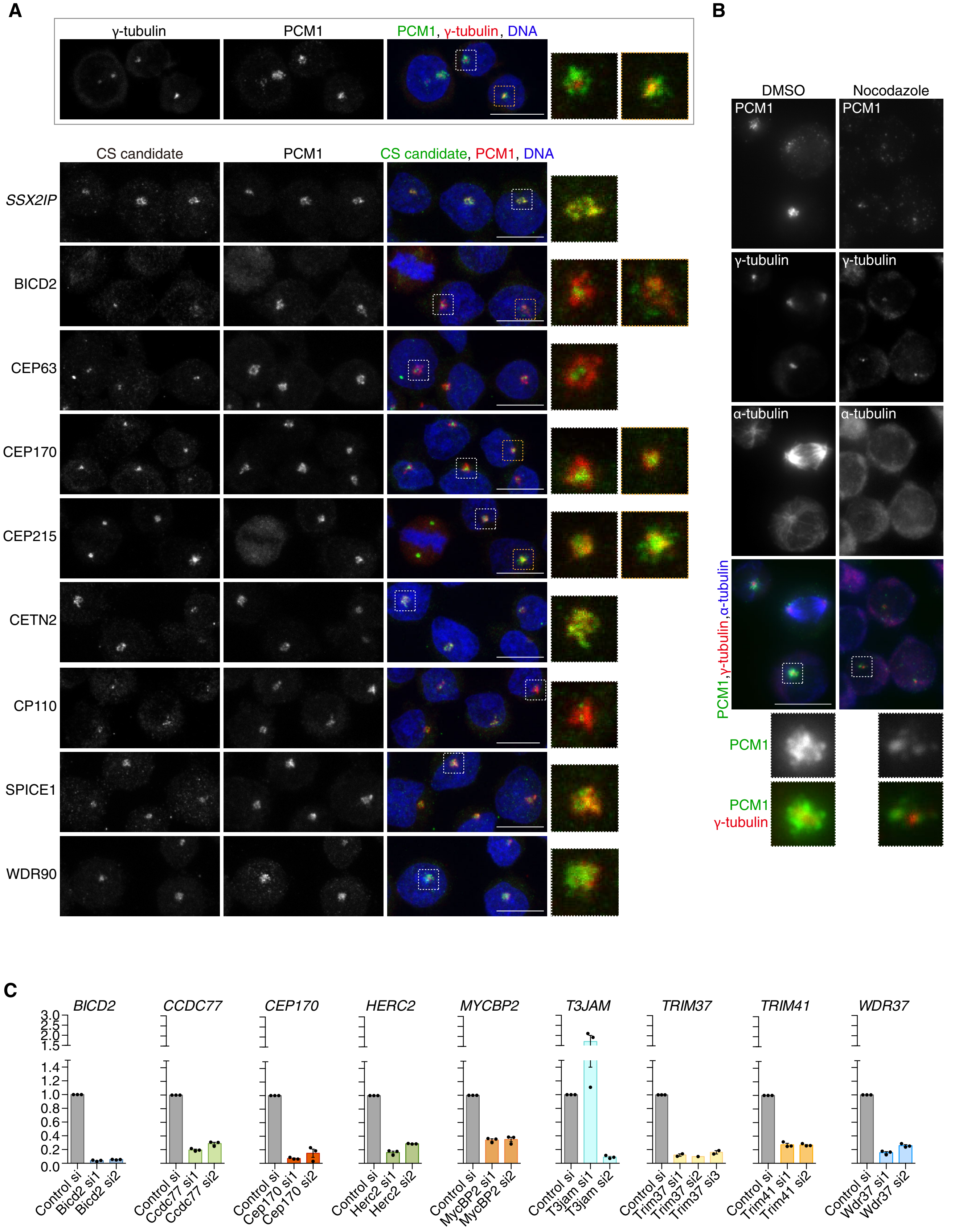

Figure EV3.

© 2019 The Authors

Figure EV4. SILAC-based quantitative MS analyses of whole-cell and centriolar satellite proteomes of $WT^{PCM1\text{-}GFP}$ and $STIL\text{-}KO^{PCM1\text{-}GFP}$ cell lines.

A Reproducibility of SILAC-WCP experiments.

B, C Reproducibility of SILAC-CS experiments. Scatterplots (in red) showing the reproducibility and quantile–quantile plots (in blue) showing similar ratio distribution between the replicates, for the GFP pull-downs (B) and the IgG CT pull-downs (C). Protein ratios of the reverse experiment have been inverted.

D Venn diagram showing the overlap between the proteins down-regulated in SILAC-CS-STIL and the centrosome proteome dataset (Jakobsen *et al*, 2011).

E Venn diagram showing the overlap between the proteins up-regulated in SILAC-CS-STIL and the centrosome proteome dataset (Jakobsen *et al*, 2011). Note that centrosomal proteins are under-represented among the up-regulated proteins.

© 2019 The Authors

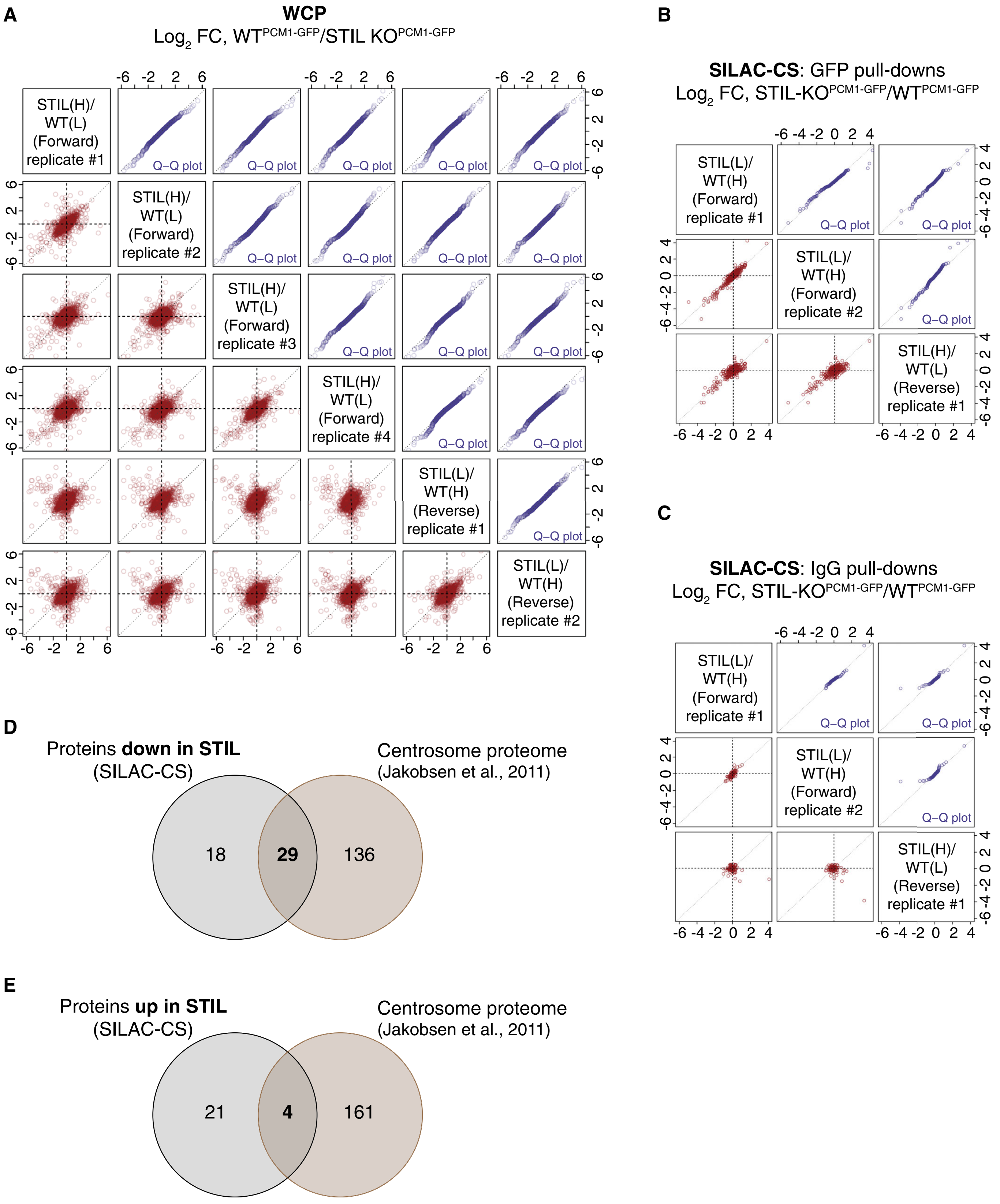
A
WCP
Log2 FC, WT PCM1-GFP/STIL KO PCM1-GFP
STIL(H)/ WT(L) (Forward) replicate #1
STIL(H)/ WT(L) (Forward) replicate #2
STIL(H)/ WT(L) (Forward) replicate #3
STIL(H)/ WT(L) (Forward) replicate #4
STIL(L)/ WT(H) (Reverse) replicate #1
STIL(L)/ WT(H) (Reverse) replicate #2
Q–Q plot
B
SILAC-CS: GFP pull-downs
Log2 FC, STIL-KO PCM1-GFP/WT PCM1-GFP
STIL(L)/ WT(H) (Forward) replicate #1
STIL(L)/ WT(H) (Forward) replicate #2
STIL(H)/ WT(L) (Reverse) replicate #1
C
SILAC-CS: IgG pull-downs
Log2 FC, STIL-KO PCM1-GFP/WT PCM1-GFP
STIL(L)/ WT(H) (Forward) replicate #1
STIL(L)/ WT(H) (Forward) replicate #2
STIL(H)/ WT(L) (Reverse) replicate #1
D
Proteins down in STIL (SILAC-CS)
Centrosome proteome (Jakobsen et al., 2011)
18
29
136
E
Proteins up in STIL (SILAC-CS)
Centrosome proteome (Jakobsen et al., 2011)
21
4
161

Figure EV4.

Figure EV5. Characterisation of PCM1-KO cell lines.

A CRISPR/Cas9 strategy to generate PCM-deficient cells. The PAM sequences are highlighted in bold. Target sequences in exons 3 and 26 are shown. Below, predicted translational products from all the sequenced variants are listed. Briefly, PCM1 variants were identified by amplifying and sequencing the regions of PCM1 cDNA targeted by the gRNAs. Amino acids deleted are represented by the dash symbol, those divergent from the control sequence are highlighted in red, whereas stop of translation is represented by the asterisk. For each clone, 10–25 bacterial colonies were sequenced. The percentage of each transcript variant/translational product is indicated at the right of each panel. For PCM1-KO 3, the PCM1 cDNA sequence was not detected.

B Representative immunofluorescence images of PCM1 control (CON 1) and KO (KO 3) cells co-stained with antibodies against PCM1 (green) and γ-tubulin (red). DNA is in blue. Images correspond to maximum intensity projections of wide-field micrograph. Scale bar: 10 μm.

C Bar chart depicts the percentage of ciliated cells in RPE-1 WT cells, control (CON) and PCM1-KO clones, after serum starvation. Cells were scored at 24, 48, 72 and 96 h after serum starvation. 200–400 cells were counted for each time-point. Data shown corresponds to a single experiment.

D Graph showing the rate of cell growth in the various genotypes. Numbers were normalised to day 0. WT: parental cell line; CON: control clone; KO: PCM1-KO clones. n = 2 replicates are plotted as mean ± SD.

 © 2019 The Authors

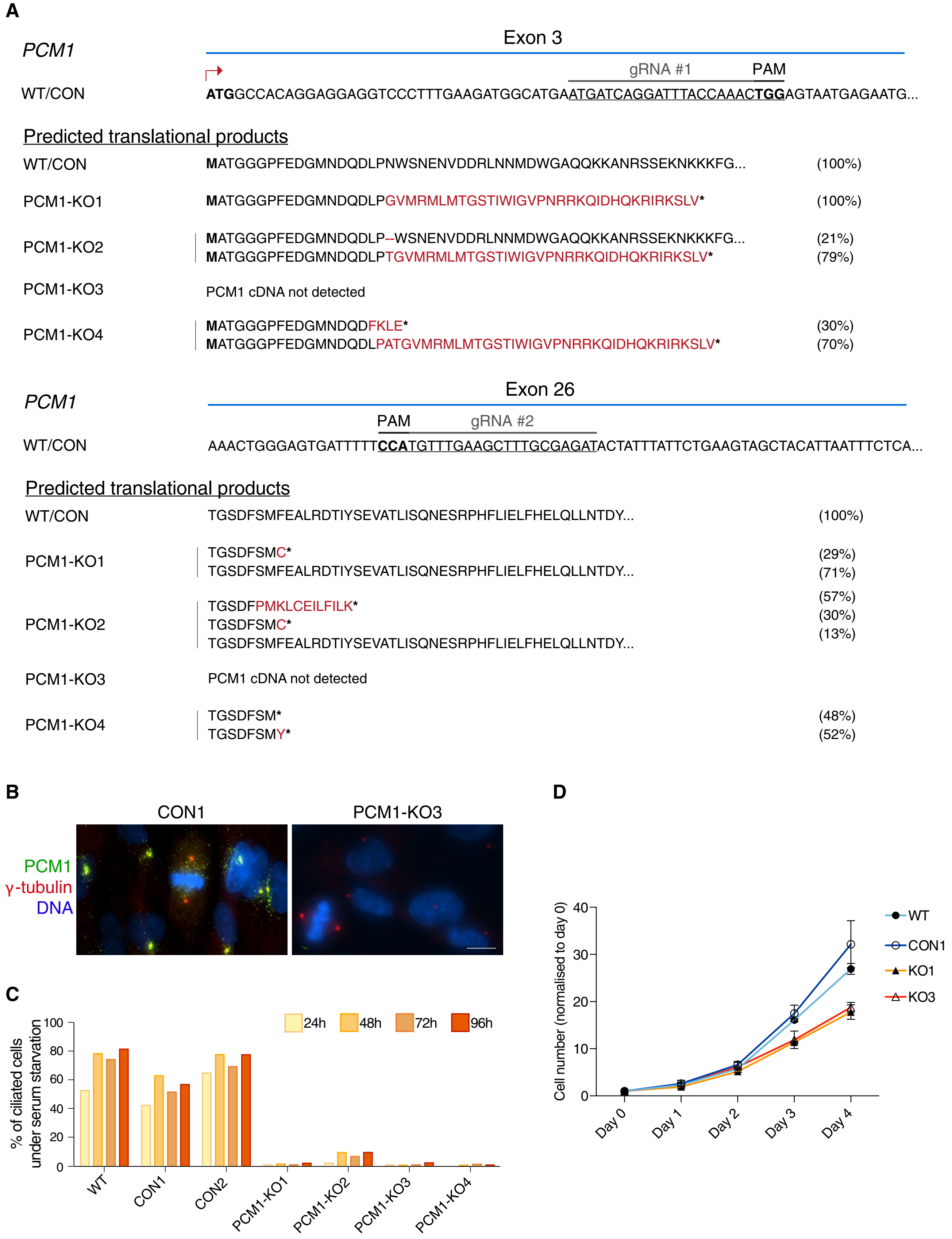
A
PCM1
Exon 3
gRNA #1
PAM
WT/CON
ATGGCCACAGGAGGAGGTCCCTTTGAAGATGGCATGAATGATCAGGATTTACCAAACTGGAGTAATGAGAATG...
Predicted translational products
WT/CON MATGGGPFEDGMNDQDLPNWSNENVDDRLNNMDWGAQQKKANRSSEKNKKKFG... (100%)
PCM1-KO1 MATGGGPFEDGMNDQDLPGVMRMLMTGSTIWIGVPNRRKQIDHQKRIRKSLV* (100%)
PCM1-KO2 MATGGGPFEDGMNDQDLP--WSNENVDDRLNNMDWGAQQKKANRSSEKNKKKFG... (21%)
MATGGGPFEDGMNDQDLPTGVMRMLMTGSTIWIGVPNRRKQIDHQKRIRKSLV* (79%)
PCM1-KO3 PCM1 cDNA not detected
PCM1-KO4 MATGGGPFEDGMNDQDFKLE* (30%)
MATGGGPFEDGMNDQDLPATGVMRMLMTGSTIWIGVPNRRKQIDHQKRIRKSLV* (70%)
PCM1
Exon 26
PAM
gRNA #2
WT/CON
AAACTGGGAGTGATTTTTCCATGTTTGAAGCTTTGCGAGATACTATTTATTCTGAAGTAGCTACATTAATTTCTCA...
Predicted translational products
WT/CON TGSDFSMFEALRDTIYSEVATLISQNESRPHFLIELFHELQLLNTDY... (100%)
PCM1-KO1 TGSDFSMC* (29%)
TGSDFSMFEALRDTIYSEVATLISQNESRPHFLIELFHELQLLNTDY... (71%)
PCM1-KO2 TGSDFPMKLCEILFILK* (57%)
TGSDFSMC* (30%)
TGSDFSMFEALRDTIYSEVATLISQNESRPHFLIELFHELQLLNTDY... (13%)
PCM1-KO3 PCM1 cDNA not detected
PCM1-KO4 TGSDFSM* (48%)
TGSDFSMY* (52%)
B
CON1
PCM1-KO3
PCM1
γ-tubulin
DNA
C
% of ciliated cells under serum starvation
100
80
60
40
20
0
24h
48h
72h
96h
WT
CON1
CON2
PCM1-KO1
PCM1-KO2
PCM1-KO3
PCM1-KO4
D
Cell number (normalised to day 0)
40
30
20
10
0
Day 0
Day 1
Day 2
Day 3
Day 4
WT
CON1
KO1
KO3

Figure EV5.

A

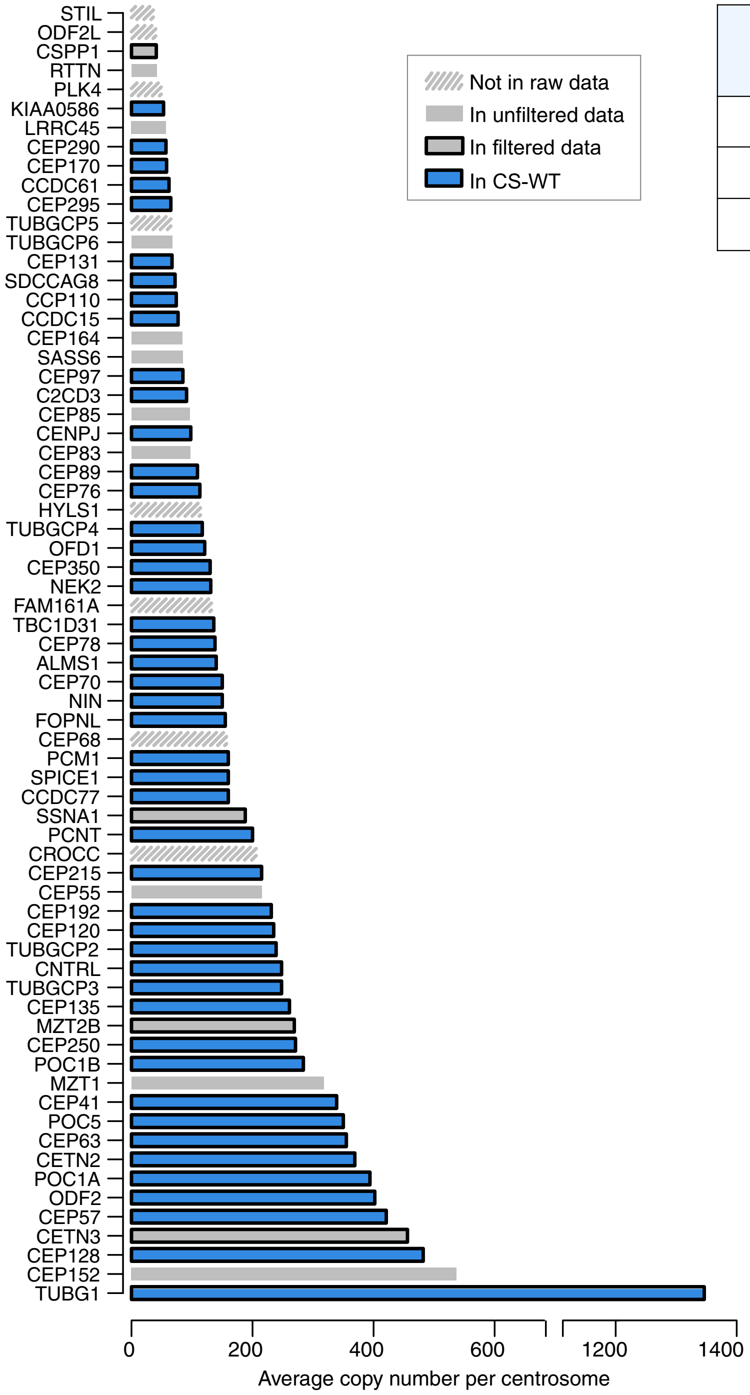

B

Proteins with known centrosomal abundance (Bauer et al., 2016)	p value (Wilcoxon rank-sum test)
Present vs. absent in Unfiltered	0.031
Present vs. absent in Filtered	0.012
Present vs. absent in CS-WT	0.045

Figure EV6. Centrosomal abundance of proteins positively correlates with their presence in the satellite proteome.

A Graph depicts relative protein abundance in centrosome preparations from KE37 cells (adapted from Fig 3, Bauer *et al*, 2016) and their status in our datasets. The categories Unfiltered and Filtered Data and CS-WT are defined in Fig 2A. Proteins absent from the chicken genome have been removed. See also Table EV4.

B To assay correlation between centrosomal abundance of proteins (from Bauer *et al*, 2016) and their presence in our satellite proteome datasets, centrosomal proteins were categorised based on their detection in our satellite proteome datasets. For each category (presence vs. absence), the abundance ranks of protein copy numbers were then compared using a two-sided Wilcoxon rank-sum test and the corresponding *P* values are depicted in the table.

 © 2019 The Authors
